# Blog and Podcast Watch: Cutaneous Emergencies

**DOI:** 10.5811/westjem.2016.11.32092

**Published:** 2017-01-19

**Authors:** Andrew Grock, Eric J. Morley, Lynn Roppolo, Jay Khadpe, Felix Ankel, Michelle Lin

**Affiliations:** * Olive View-UCLA Medical Center, Department of Emergency Medicine, Sylmar, California; † University of Southern California Keck School of Medicine and Medical Center, Department of Emergency Medicine, Los Angeles, California; ‡ Stony Brook School of Medicine, Department of Emergency Medicine, Stony Brook, New York; § University of Texas Southwestern, Department of Emergency Medicine, Dallas, Texas; ¶ University of Florida College of Medicine, Department of Emergency Medicine, Jacksonville, Florida; || HealthPartners Institute, Health Professions Education, Bloomington, Minnesota; # University of Minnesota Medical School, Department of Emergency Medicine, Minneapolis, Minnesota; ** University of California San Francisco, Department of Emergency Medicine, San Francisco, California

## Abstract

**Introduction:**

The *WestJEM* Blog and Podcast Watch presents high quality open-access educational blogs and podcasts in emergency medicine (EM) based on the ongoing Academic Life in Emergency Medicine (ALiEM) Approved Instructional Resources (AIR) and AIR-Professional series. Both series critically appraise resources using an objective scoring rubric. This installment of the Blog and Podcast Watch highlights the topic of cutaneous emergencies from the AIR series.

**Methods:**

The AIR series is a continuously building curriculum that follows the Council of Emergency Medicine Residency Directors (CORD) annual testing schedule. For each module, relevant content is collected from the top 50 most accessed sites per the Social Media Index published within the previous 12 months and scored by eight board members using five equally weighted measurement outcomes: Best Evidence in Emergency Medicine (BEEM) score, accuracy, educational utility, evidence based, and references. Resources scoring ≥30 out of 35 available points receive an AIR label. Resources scoring 27–29 receive an “honorable mention” label, if the editorial board agrees that the post is accurate and educationally valuable.

**Results:**

A total of 35 blog posts and podcasts were evaluated. None scored ≥30 points necessary for the AIR label, although four honorable mention posts were identified. Key educational pearls from these honorable mention posts are summarized.

**Conclusion:**

This Blog and Podcast Watch series is based on the AIR and AIR-Pro series, which attempts to identify high quality educational content on open-access blogs and podcasts. This series provides an expert-based, post-publication curation of educational social media content for EM clinicians with this installment focusing on cutaneous emergencies.

## BACKGROUND

Despite the rapid rise of social media educational content available through blogs and podcasts in emergency medicine (EM),^1^ identification of quality resources for educators and learners has only minimally progressed.^2^–^4^ In 2008, the Accreditation Council for Graduate Medical Education endorsed a decrease in synchronous conference experiences for EM residency programs by up to 20% in exchange for asynchronous learning termed Individualized Interactive Instruction (III).^5^ Residency programs, however, are often unsure how to identify quality online resources specifically for asynchronous learning and III credit.

To address this need, the Academic Life in Emergency Medicine (ALiEM) Approved Instructional Resources (AIR) Series and AIR-Pro Series were created in 2014 and 2015, respectively, to help EM residency programs identify quality online content specifically on social media.^6^,^7^ Using an expert-based, crowd-sourced approach, these two programs identify trustworthy, high quality, educational blog and podcast content. This Blog and Podcast Watch series presents annotated summaries written by the editorial board from the AIR and AIR-Pro Series.

This installment from the AIR Series summarizes the highest scoring social media educational resources on cutaneous emergencies.

## METHODS

### Topic Identification

The AIR series is a continuously building curriculum based on the CORD testing schedule (http://www.cordtests.org/).

### Inclusion and Exclusion Criteria

A search of the top 50 most frequently visited sites per the Social Media Index^8^ was conducted in March 2016 for resources relevant to cutaneous emergencies, published within the previous 12 months. Methodology details for inclusion, exclusion, scoring criteria, and data analyses are summarized in the original AIR publication.^6^

### Scoring

Extracted posts were scored by eight reviewers from the AIR Editorial Board, which is comprised of EM core faculty from various U.S. medical institutions. The scoring instrument contains five measurement outcomes using seven-point Likert scales: Best Evidence in Emergency Medicine (BEEM) score, accuracy, educational utility, evidence based, and references (Table).^6^,^9^

### Data Analysis

Resources with a mean score of ≥ 30 points (out of 35) are awarded the AIR label. Resources with a mean score of 27–29, deemed accurate and educationally valuable by the reviewers, receive the “honorable mention” label.

## RESULTS

A total of 35 blog posts and podcasts were initially collected and reviewed. None scored ≥30 points necessary for the AIR label, although four honorable mention posts were identified. Key educational pearls from these honorable mention AIR posts are described.

### AIR Honorable Mention Content

#### 1. Hayes B, Awad N, Heil E. Sulfamethoxazole-Trimethoprim for Skin and Soft Tissue Infections: 1 or 2 Tablets BID? *Academic Life in Emergency Medicine*. (February 16, 2015) https://www.aliem.com/2015/sulfamethoxazole-trimethoprim-ssti-1-2-tablets-bid/

Sulfamethoxazole-trimethoprim (SMX-TMP) is recommended by the 2014 Infectious Diseases Society of America guidelines for purulent, suspected methicillin-resistant *S. aureus* (MRSA) skin and soft tissue infections (SSTIs). This post compares the evidence for one versus two double strength SMX-TMP tablets twice a day.

Take-home points**:** Two studies are discussed including a prospective evaluation of patients with confirmed MRSA SSTIs and a retrospective study of 106 patients hospitalized for cellulitis with and without abscess. The first study found no difference in clinical resolution of the infection between the two doses, while the second study showed increased clinical failure in morbidly obese patients taking one double-strength tablet SMX-TMP per dose. While not shown to be helpful in most patients, the increased dose of two tablets twice a day may be appropriate for patients with obesity, immunosuppression, and trauma-induced SSTIs. However, this increased dose may be associated with increased adverse effects including hyperkalemia.

#### 2. Schneider E. SGEM#110: I saw the signs of angioedema. Skeptics Guide to Emergency Medicine. (March 7, 2015) http://thesgem.com/2015/03/sgem110-i-saw-the-signs-of-angioedema/

Non-allergic angioedema from angiotensin converting enzyme inhibitors (ACE-I) is thought to be bradykinin-mediated and is therefore resistant to standard anaphylaxis therapies of epinephrine, antihistamines, and corticosteroids. This post reviews a randomized control trial of icatibant, a bradykinin receptor antagonist, for the treatment of ACE-I associated angioedema.

Take-home points: The study enrolled 27 emergency department patients who presented with angioedema of the upper aerodigestive tract and were taking an ACE-I. The investigators compared icatibant 30 mg to the standard intravenous therapy of prednisolone 500 mg (corticosteroid) plus clemastine 2 mg (antihistamine and anticholinergic). The primary outcome showed that the icatibant group had a significantly shorter time to complete resolution of symptoms (8 vs. 27.1 hours). The icatibant group also had a higher proportion of complete resolution of symptoms at four hours (38% vs. 0%) and a faster time to onset of symptom relief (2 vs. 11.7 hours).

There were several limitations noted including small sample size, no documentation of consecutive enrollment, lack of a blinded study design, and funding provided by the pharmaceutical company. Additionally, all patients enrolled were Caucasian even though ACE-I angioedema is five times more common in patients of African descent. The standard care group did not include therapies, such as epinephrine or fresh frozen plasma, which may have limited the results. Patient-oriented outcomes such as mortality, need for intubation, and cost were not studied. Overall, icatibant appears to be effective for the treatment of ACE-I angioedema; however, given its cost of $5,000 – $10,000 it should be reserved for the more severe cases involving airway compromise.

#### 3. Long B. The emergency medicine approach to vasculitides. EM Docs. (June 12, 2015) http://www.emdocs.net/the-emergency-medicine-approach-to-vasculitides/

Systemic vasculitides are chronic, inflammatory, autoimmune disorders with multi-organ pathology secondary to inflammatory damage to blood vessels. This post provides an overview of emergent complications.

Take-home points: In the acute setting, beware of both infectious etiologies that require antibiotics as well as flares of rheumatic diseases that require high-dose steroids. Consultations with rheumatologists and intensivists are often indicated. Systemic lupus erythematosus (SLE) is specifically discussed and, of note, presents most commonly with rash, mucositis, and arthritis. The diagnosis of SLE is based on having ≥4 of the 11 diagnostic criteria: malar rash, discoid rash, photosensitivity, oral ulcers, non-erosive arthritis, serositis, renal disease, neurologic disorders, two or more hematologic cell lines decreased, positive anti-nuclear antibody, and another positive SLE antibody (anti-DNA, Anti-Sm, or antiphospholipid).

Vasculidites adversely affect numerous organ systems that emergency physicians should be aware of. Pulmonary complications can include interstitial fibrosis, pulmonary hypertension and diffuse alveolar hemorrhage. Thromboembolic complications can affect multiple systems resulting in such conditions as myocardial infarction, deep vein thrombosis, pulmonary embolism, renal vascular thrombosis, mesenteric ischemia, and cerebral vascular occlusion. Common cutaneous pathology includes erythema nodusum, palpable purpura from Henoch-Schonlein purpura, oral and genital ulcers of Behcet’s disease, and the malar and discoid lesions of SLE.

#### 4. Smith B. UOTW #66. Ultrasound of the Week. (January 7, 2016) http://www.ultrasoundoftheweek.com/uotw-66/

Often it is difficult to clinically distinguish cellulitis from a subcutaneous abscess. This post reviews the instructions for and utility of ultrasonography in differentiating between these two pathologies.

Take-home points: Ultrasound often changes gestalt management in differentiating between cellulitis with or without an underlying abscess. In cellulitis alone, ultrasonography often shows a cobblestone pattern. In subcutaneous abscesses, a hypo-echoic fluid collection is seen. Because ultrasonographic compression improves the sensitivity to detect subcutaneous fluid, by inducing fluid/pus movement or swirling, it should be performed every 1–2 cm throughout the area of cellulitis.

## CONCLUSION

The Blog and Podcast Watch series serves to identify educational quality blogs and podcasts for EM clinicians through its expert panel using an objective scoring instrument. These social media resources are currently curated in the ALiEM AIR and AIR-Pro Series, originally created to address EM residency needs. These resources are herein shared and summarized to help clinicians filter the rapidly published multitude of blog posts and podcasts. One of the limitations is that the search only includes content produced within the preceding12 months from the top 50 Social Media Index sites. While these lists are by no means a comprehensive analysis of the entire Internet for these topics, this series provides a post-publication curation and accreditation of recent high quality, educational social media content for the EM clinician.

## Figures and Tables

**Table:** Approved Instructional Resources (AIR) scoring instrument for blog and podcast content with the maximum score being 35 points.

Tier 1: BEEM rater scale	Score	Tier 2: content accuracy	Score	Tier 3: educational utility	Score	Tier 4: evidence based medicine	Score	Tier 5: referenced	Score
Assuming that the results of this article are valid, how much does this article impact on EM clinical practice?		Do you have any concerns about the accuracy of the data presented or conclusions of this article?		Are there useful educational pearls in this article for senior residents?		Does this article reflect evidence based medicine (EBM)?			Are the authors and literature clearly cited?
Useless information	1	Yes, many concerns from many inaccuracies	1	Not required knowledge for a competent EP	1	Not EBM based, only expert opinion	1	No	1
Not really interesting, not really new, changes nothing	2		2		2		2		2
Interesting and new, but doesn’t change practice	3	Yes, a major concern about few inaccuracies	3	Yes, but there are only a few (1–2) educational pearls that will make the EP a better practitioner to know or multiple (>=3) educational pearls that are interesting or potentially useful, but rarely required or helpful for the daily practice of an EP.	3	Minimally EBM based	3		3
Interesting and new, has the potential to change practice	4		4		4		4	Yes, authors and general references are listed (but no in-line references)	4
New and important: this would probably change practice for some EPs	5	Minimal concerns over minor inaccuracies	5	Yes, there are several (>=3) educational pearls that will make the EP a better practitioner to know, or a few (1–2) every competent EP must know in their practice	5	Mostly EBM based	5		5
New and important: this would change practice for most EPs	6		6		6		6		6
This is a “must know” for EPs	7	No concerns over inaccuracies	7	Yes, there are multiple educational pearls that every competent EP must know in their practice	7	Yes exclusively EBM based	7	Yes, authors and in-line references are provided	7

*BEEM*, best evidence in emergency medicine; *EP*, emergency physician; *EBM,* evidence-based medicine.
